# *BMAL1* Genetic Variation in Metabolic and Mental Health

**DOI:** 10.1192/bjo.2024.73

**Published:** 2024-08-01

**Authors:** Hamza H Daudali, Breda Cullen, Nicholas Graham, Joey Ward, Rona Strawbridge

**Affiliations:** 1 Behavioural Epidemiology and Genetics, School of Health and Wellbeing, University of Glasgow, Glasgow, United Kingdom; 2 Cardiovascular Medicine Unit, Department of Medicine, Karolinska Institute, Stockholm, Sweden; 3 Health Data Research UK, Glasgow, United Kingdom

## Abstract

**Aims:**

Epidemiological studies have previously shown a link between cardiometabolic disease and severe mental illness. The extent and mechanisms behind this link are poorly understood currently but links to impairments in the stress response and cortisol regulation have been thought to play a significant role. *BMAL1* is a circadian rhythm regulation gene found on chromosome 11 which has been associated with a variety of pro-inflammatory states as well as conditions such as depression, schizophrenia, type 2 diabetes mellitus and myocardial infarction. Our study aimed to investigate the genetic structure of the *BMAL1* gene locus and its associations with both cardiometabolic and psychiatric traits and conditions.

**Methods:**

We used genetic data from the UK Biobank which recruited ~500,000 participants. Of these we used a population of ~430,000 self-reported white British participants and data from a variety of questionnaires and investigations looking at severe mental illness and cardiometabolic traits. We performed association analyses using Plink 1.07 with Bonferroni correction being performed for multiple testing using a number of genetic variants. Our threshold for significance was defined as a p-value < 5.35 × 10^−5^. Conditional analysis was then performed to identify if there were multiple independent signals for each phenotype.

**Results:**

*BMAL1* variants were associated with BMI, diastolic, systolic blood pressure, waist-hip ratio and neuroticism score, and risk of anhedonia, major depressive disorder and risk-taking behaviour. Multiple significant independent signals were identified for BMI and waist-hip ratio. Linkage disequilibrium (LD) analysis showed significant coinheritance of specific traits which could suggest a role for *BMAL1* and the encoded protein as a link between cardiometabolic and mental health traits.

**Conclusion:**

This is the first study that systematically investigated associations between the *BMAL1* locus across a variety of different mental and cardiometabolic phenotypes in a population-level cohort. Our study has shown that there is a link between the *BMAL1* locus and both cardiometabolic and mental health phenotypes. Further research is required to investigate the exact biological mechanism by which *BMAL1* connects severe mental illness and cardiometabolic disease.

